# Impact of D-dimer for pathologic differentiation on transesophageal echocardiography in embolic stroke of undetermined source: a single-center experience

**DOI:** 10.1186/s12883-022-02867-z

**Published:** 2022-09-08

**Authors:** Kenichiro Hira, Yuji Ueno, Masao Watanabe, Hideki Shimura, Naohide Kurita, Nobukazu Miyamoto, Haruna Haginiwa, Kazuo Yamashiro, Nobutaka Hattori, Takao Urabe

**Affiliations:** 1 Department of Neurology, Juntendo University Urayasu Hospital, 2-1-1 Tomioka, Urayasu, Chiba 279-0021 Japan; 2 Department of Neurology, Juntendo University School of Medicine, 2-1-1 Hongo, Bunkyo-ku, Tokyo, 113-8421 Japan

**Keywords:** Embolic stroke of undetermined source, Transesophageal echocardiography, Patent foramen ovale, D-dimer

## Abstract

**Background:**

Embolic stroke of undetermined source (ESUS) encompasses diverse embologenic mechanisms, which transesophageal echocardiography (TEE) is critical to detect. Specific markers related to each embolic source in ESUS is not fully studied. We focused on D-dimer levels, and explored the association of D-dimer with potential embolic sources (PES) identified on TEE in ESUS.

**Methods:**

Consecutive patients with ESUS were included in this study. Clinical characteristics including D-dimer levels were compared between ESUS patients with and without TEE, and among none of, one, and at least two PES in ESUS patients undergoing TEE. Factors related to elevation of D-dimer were analyzed.

**Results:**

A total of 211 patients (age, 69.3 ± 13.2 years; 149 males) with ESUS were enrolled. Of these, 115 received TEE, displaying significantly younger age and lower D-dimer levels than patients without TEE (*P* < 0.05), and 20 (17%), 61 (53%), and 34 (30%) patients were classified into none of, one, and ≥ two PES, respectively. On multiple logistic regression analysis, D-dimer levels were related to one PES (odds ratio [OR]: 9.01; 95% confidence interval [CI]: 1.00–81.51; *P* = 0.050) and PES ≥ two (OR: 9.76; 95% CI: 1.07–88.97; *P* = 0.043). Right-to-left shunt (RLS) with deep venous thrombosis (DVT)(OR: 13.94; 95% CI: 1.77–109.99; *P* = 0.012) and without DVT (OR: 3.90; 95% CI: 1.20–12.70; *P* = 0.024) were associated with elevation of D-dimer.

**Conclusions:**

D-dimer levels were higher in patients with PES. Among PES, RLS, with and without DVT, were associated with increase of D-dimer in ESUS.

## Background

Ischemic stroke with undetermined etiologies is known as cryptogenic stroke, and has a frequency of 20–30% [1–3]. The majority of cryptogenic stroke occurs through embolism, and the term embolic stroke of undetermined source (ESUS) was advocated in 2014 [4]. The recurrence rate of ESUS and cryptogenic stroke is the second highest following to cardioembolism among all ischemic stroke subtypes [5, 6].

Transesophageal echocardiography (TEE) is a useful modality to detect potential embolic sources (PES) such as patent foramen ovale (PFO), atrial septal aneurysm (ASA), and complex aortic plaques, while spontaneous echo contrast (SEC) on TEE can indicate the presence of paroxysmal atrial fibrillation (AF), especially in ESUS [7, 8]. Although TEE is not included in the diagnostic criteria of ESUS, recent studies performed TEE for ESUS patients and clarified the incidence of each embologenic disease in ESUS [9, 10]. TEE is semi-invasive and cannot be performed in all ESUS patients, thus, evidence regarding potential embolic diseases in ESUS has not been fully elucidated. Although two large-scale clinical trials, the New Approach Rivaroxaban Inhibition of Factor Xa in a Global Trial vs. ASA to Prevent ESUS (NAVIGATE-ESUS) trial and the Randomized, double-blind, Evaluation in secondary Stroke Prevention comparing the EfficaCy and safety of the oral Thrombin inhibitor dabigatran etexilate vs. acetylsalicylic acid in patients with Embolic Stroke of Undetermined Source (RE-SPECT ESUS) trial did not show any efficacies of direct oral anticoagulants (DOACs) in the secondary prevention of ESUS. A subanalysis of the NAVIGATE ESUS demonstrated that rivaroxaban significantly reduced stroke recurrence in ESUS patients with PFO and an LA diameter > 4.6 cm [11–14]. In the previous ESUS registry, more than two thirds of ESUS patients have at least one potential embolic source [15]. It is suggested that underlying embolic etiologies may be critical to determine the effect of DOACs in ESUS.

An emerging data indicated that coagulation markers including D-dimer have been critical to stratify comorbidities such as cancer, venous thromboembolism, and coagulopathies in cryptogenic stroke [16]. However, association of underlying embologenic diseases on TEE with coagulation markers is yet to be elucidated. Meanwhile, TEE is carried out for limited ESUS patients according to the stroke physicians’ patient selection due to such as stroke severity in real-world clinical practice, and no evidence to determine the factors indicating TEE for ESUS is available. In the present study, we focused on the association of PES demonstrated on TEE with D-dimer, and elucidated the clinical significance of D-dimer levels between patients with and without TEE, and existence and complexity of PES on TEE in ESUS.

## Material and methods

### Patients

This case series was based on a retrospective analysis of data acquired from the stroke registry of patients admitted to the Department of Neurology at Juntendo University Urayasu Hospital during the study period from October 1, 2013 to March 31, 2018. Stroke severity was assessed by the National Institute Health of Stroke Scale (NIHSS) score on admission. All patients with ischemic stroke underwent blood collection, chest X-ray, 12-lead ECG, brain magnetic resonance imaging (MRI), MR angiography (MRA), brain computerized tomography, carotid ultrasonography, Holter ECG monitoring for 24 h, cardiac ECG monitoring ≥24 h, and transthoracic echocardiography on admission. From these investigations, we excluded cardioembolism, large artery atherosclerosis, small artery occlusion, and other determined stroke etiology according to the Trial of ORG 10172 in Acute Stroke Treatment [17], and patients categorized to stroke of undetermined etiology and who fulfilled the original diagnostic criteria of ESUS by Hart et al. were included in our analyses [4]. Performance of TEE was decided in the conference by three experienced stroke physicians (K.H, M.W, T.U) considering the safety and avoiding complications according to the American Society of Echocardiography Guidelines and Standards [18]. ESUS patients were classified into two groups based on the performance of TEE. Furthermore, patients with TEE were divided into none of, one, and two PES. We used clinical information obtained from medical records, and the need to obtain written informed consent for publication from each patient was therefore waived in this retrospective study. The present study was approved by the ethics committees of Juntendo University Urayasu Hospital. This study was conducted in accordance with the Declaration of Helsinki.

### Risk factors

We defined atherosclerotic vascular risk factors as follows: 1) hypertension [systolic blood pressure >  140 mmHg or diastolic blood pressure > 90 mmHg (in subacute phase; ≥14 days from admission), history of hypertension or using antihypertensive agents]; 2) diabetes mellitus (use of oral hypoglycemic agents or insulin, or hemoglobin A1c ≥6.5%); 3) dyslipidemia [using antihyperlipidemic agents, serum low-density lipoprotein cholesterol (LDL-C) ≥140 mg/dl, high-density lipoprotein cholesterol (HDL-C) < 40 mg/dl, or triglycerides ≥150 mg/dl]; 4) Chronic kidney disease (CKD) [an estimated glomerular filtration rate (eGFR) < 60 ml/min/1.73 m^2^ calculated by the following equation for Japanese adults approved by the Japanese Society of Nephrology: eGFR = 194*serum creatinine^-1.094^*Age^-0.287^*0.739 (if female)]; 5) malignancy (history of malignancy and active cancer); 7) smoking (current); 8) coronary artery disease (defined as a history of angina pectoris or myocardial infarction); and 9) history of ischemic stroke [19, 20].

### Radiological investigations

MRI was performed using a 1.5-Tesla MR scanner equipped with single shot echo planar imaging (EXELART Vantage; Toshiba, Tokyo, Japan) and included diffusion-weighted imaging (DWI), T2-weighted images, fluid attenuation inversion recovery (FLAIR), and MRA. We diagnosed brain infarction by focal hyperintensity that was judged not attributable to normal anisotropic diffusion or magnetic susceptibility artifact. Total imaging time was approximately 20 min. A standard DWI sequence (repetition time (TR)/echo time (TE) = 7000/120, 240 × 260-mm field of view, 192 × 198 matrix, 5-mm section thickness, 1-mm intersection gap), FLAIR sequence (TR/TE = 7000/105, 220 × 230-mm field of view, 192 × 320 matrix, 5-mm section thickness, 1-mm intersection gap, two signals acquired), and MRA sequence (TR/TE = 30/6.8, 240 × 260-mm field of view, 192 × 256 matrix, 1-mm section thickness, 1-mm intersection gap) were performed. Cerebral microbleeds were achieved by gradient-echo T2*-weighted MRI sequences (TR/TE = 696/15, 220 × 230-mm field of view, 192 × 320 matrix, 5-mm section thickness, 1-mm intersection gap, two signals acquired). Diameters of infarct areas were measured on axial DWI slices, and size, location, and number of infarcts were analyzed. According to previous studies, periventricular hyperintensity (PVH) and deep and subcortical white matter hyperintensity (DSWMH) were defined as high-intensity lesions on axial FLAIR images [21]. Stenosis of intracranial arteries, including the anterior, middle, and posterior cerebral arteries and the vertebral and basilar arteries, that were not the arteries of cerebral infarction was diagnosed as greater than 50% stenosis on MRA.

### TEE study

TEE was performed using a Vivid S6 system equipped with a multiplane 7 MHz transducer (GE Medical Systems, Tirat Carmel, ISRAEL) according to a previous protocol [9, 22]. During TEE examinations, the patients were awake, and lidocaine spray, but no premedication was used. PFO was evaluated by injecting agitated saline and having patients perform the Valsalva maneuver. The numbers of microbubbles with and without contrast agents were compared. The number of microbubbles that moved from the right atrium to the left atrium through the foramen was also counted. PFO was assessed when microbubbles were visualized in the left atrium during the Valsalva maneuver [19]. ASA was diagnosed when the atrial septum extended at least 10 mm into the left or right atrium, or had a sum of total excursion into the left or right atrium of ≥15 mm [23]. Flow velocities in the left atrial appendage (LAA) were measured, and presence of thrombus in the LAA were assessed. Aortic arch plaque thickness was measured, and ≥ 4 mm or plaques with ulceration or mobile components were considered as complex aortic plaques [19]. The examinations were performed by experienced sonographers (K.H., and M.W.) and video recorded.

### Definition of potential embolic sources on TEE

We classified PES based on TEE findings into the following classification: 1) right-to-left shunt (RLS) including PFO; 2) ASA; 3) complex aortic plaques; and 4) LAA dysfunction (LAA flow velocities < 0.2 m/s, or presence of LAA thrombus) or SEC [24]. In patients with RLS, deep venous thrombosis (DVT) was assessed by duplex ultrasonography.

### Biochemical blood tests

Laboratory data included LDL-C, HDL-C, hemoglobin bA1c, eGFR, brain natriuretic peptide (BNP), and D-dimer levels. Blood collection was performed when the patient was transferred to emergency departments on admission, or immediately after referral to physicians in our department when patients developed ischemic stroke during hospitalization, before any stroke treatments.

### Statistical analysis

Numerical values are reported as means ± standard deviation. Baseline characteristics, laboratory data, and radiological observations were compared between the patients with TEE and patients without TEE, and among none of, one, and ≥ two PES. Data were analyzed using the chi-squared test for categorical variables, and the Mann–Whitney and Kruskal-Wallis tests for nonparametric analyses. All variables with a value of *P* < 0.1 on univariate analyses were entered into the multiple logistic regression analysis to identify independent factors for performing TEE. Factors related to performance of TEE, presence of PES, and elevation of D-dimer levels were investigated by the multiple logistic regression. A *P* value < 0.05 was considered significant. All data were analyzed using SPSS for Macintosh version 26.0 software (SPSS, Chicago, IL).

### Data availability

The data that support the findings of this study are available from the corresponding author upon reasonable request. Retrospectively collected data from Juntendo University Urayasu Hospital was pooled with the use of a standardized form.

## Results

**Fig. 1:**
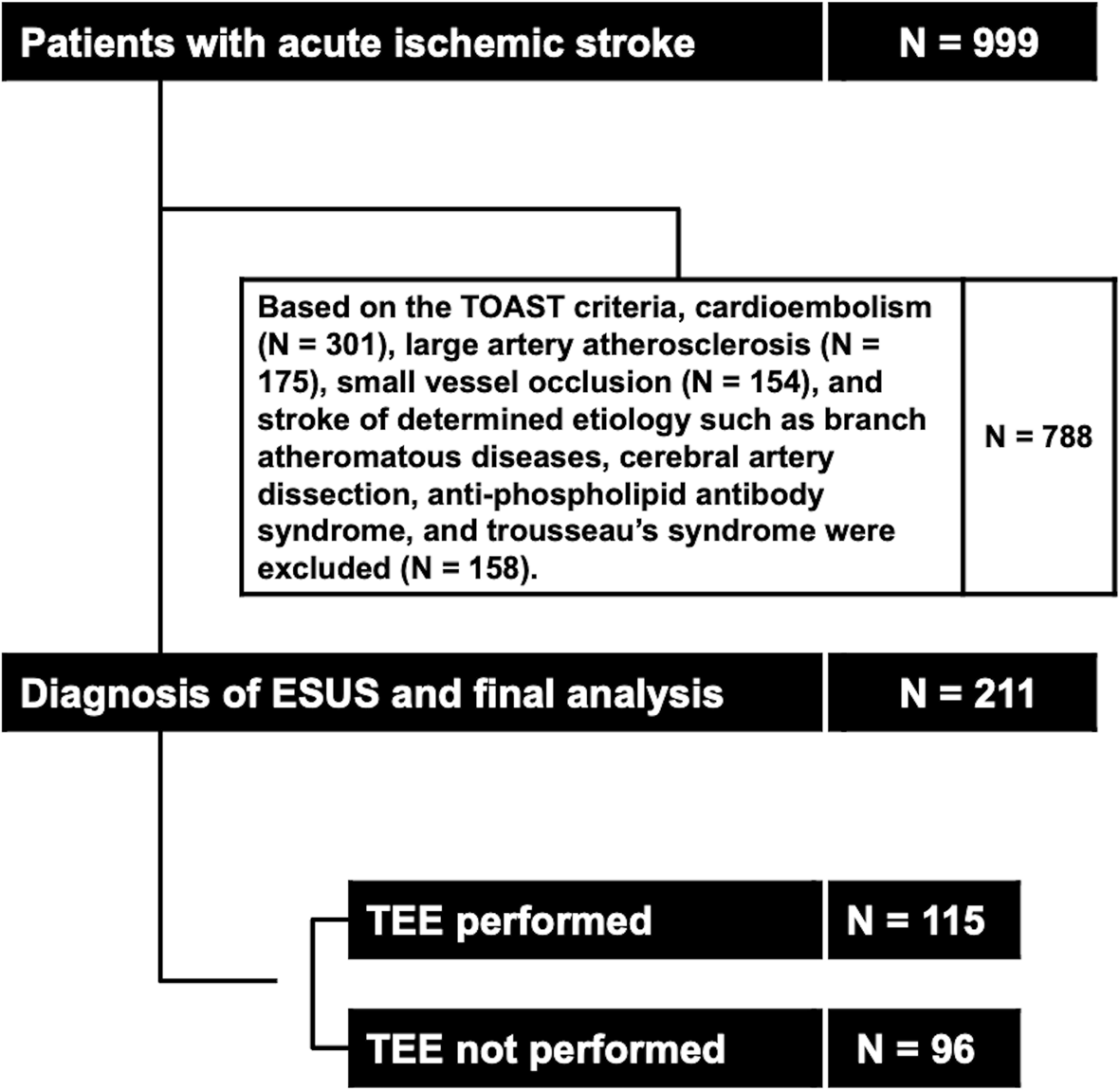
Flow diagram of the current study. TOAST, Trial of Org 10,172 in Acute Stroke Treatment; ESUS, embolic stroke of undetermined source; TEE, transesophageal echocardiography

A total of 999 patients with ischemic stroke were admitted during the study period. Of these, 301 patients had cardioembolism, 175 had large artery atherosclerosis, 154 had small vessel occlusion, and 158 patients had stroke with other determined etiology such as branch atheromatous diseases, cerebral artery dissection, and anti-phospholipid antibody syndrome. All of these patients were excluded. Finally, 211 patients (age, 69.3 ± 13.2 years; 149 males) met the diagnostic criteria of ESUS, and were included in the final analysis. Of the 211 patients, 115 patients (55%) underwent TEE (With TEE group, Fig. 1).

### Difference of clinical characteristics between ESUS patients with and without TEE

Table 1 lists the baseline characteristics for all patients, and each group, with TEE or without TEE. Patients with TEE were younger than patients without TEE (66.6 ± 12.0 years vs. 72.5 ± 13.9 years, *P* < 0.001). Male subjects were more common in patients with TEE than patients without TEE (77% vs. 63%, *P* = 0.018). No significant differences were observed in atherosclerotic vascular risk factors between the two groups. The NIHSS score was lower in patients with TEE than in patients without TEE (3.9 ± 5.2 vs. 7.4 ± 7.2, *P* < 0.001). In laboratory data, levels of BNP were higher in in the patients with TEE group (110.1 ± 341.1 vs. 93.0 ± 137.6 pg/ml, *P* = 0.046), while D-dimer levels were higher in the patients without TEE group (1.2 ± 1.3 vs. 2.9 ± 5.0 μg/ml, *P* < 0.001). In radiological observations, the number of patients with a grade of PVH and DSWMH ≥2 was lower in the patients with TEE group than in the patients without TEE group (28% vs. 45%, *P* = 0.010; 23% vs. 44%, *P* = 0.002; respectively). Age, male gender, CKD, NIHSS score, hemoglobin A1c, BNP, and D-dimer levels, cerebral microbleeds, and PVH and DSWMH ≥2 grade were entered into multiple logistic regression analysis to determine independent factors linked with ESUS patients who underwent TEE. Table 2 shows that NIHSS score (Odds ratio, [OR]: 0.93; 95% confidence interval [CI]: 0.88–0.98; *P* = 0.008) and D-dimer levels (OR: 0.84; 95% CI: 0.70–1.00; *P* = 0.048) were independently associated with ESUS patients with TEE.

**Table 1:** Baseline characteristics and Laboratory and MRI findings according to the performance of TEE in ESUS

	Total	TEE examination		
Variables		With	Without	*P*
	*N* = 211	*N* = 115, 55%	*N* = 96, 45%	
**Demographics**				
Age, mean ± SD	69.3 ± 13.2	66.6 ± 12.0	72.5 ± 13.9	< 0.001
Male gender, n (%)	149 (71)	89 (77)	60 (63)	0.018
Hypertension, n (%)	150 (71)	83 (72)	67 (70)	0.704
Diabetes mellitus, n (%)	86 (41)	52 (45)	34 (35)	0.149
Dyslipidemia, n (%)	155 (74)	89 (78)	66 (69)	0.157
Smoking, n (%)	54 (26)	30 (26)	24 (25)	0.857
Chronic kidney disease, n (%)	66 (31)	30 (26)	36 (38)	0.075
Coronary artery disease, n (%)	6 (3)	4 (3)	2 (2)	0.539
Malignant tumor, n (%)	13 (6)	6 (5)	7 (7)	0.533
History of cerebral infarction, n (%)	40 (19)	20 (17)	20 (21)	0.525
**NIHSS on admission**	5.5 ± 6.5	3.9 ± 5.2	7.4 ± 7.2	< 0.001
**Laboratory findings**				
LDL-C, mg/dl, a	112.9 ± 40.6	114.5 ± 36.3	110.8 ± 45.5	0.256
HDL-C, mg/dl, a	52.8 ± 15.9	51.5 ± 14.9	54.5 ± 16.9	0.217
eGFR, ml/min	69.2 ± 24.9	72.0 ± 25.4	65.7 ± 24.0	0.127
Hemoglobin A1c, %, b	6.2 ± 1.1	6.3 ± 1.1	6.1 ± 1.0	0.099
BNP, pg/ml, c	100.9 ± 265.9	110.1 ± 341.1	93.0 ± 137.6	0.046
D-dimer, μg/ml, d	2.7 ± 11.4	1.2 ± 1.3	2.9 ± 5.0	< 0.001
**MRI findings, n (%)**				
Multiple lesions	116 (55)	66 (57)	50 (52)	0.44
Lesion size > 30 mm in diameter	69 (33)	38 (33)	31 (32)	0.908
Cortical infarction	167 (79)	93 (81)	74 (77)	0.501
Cerebral microbleeds	43 (20)	18 (16)	25 (26)	0.062
PVH, grade ≥ 2	75 (36)	32 (28)	43 (45)	0.01
DSWMH, grade ≥ 2	69 (33)	27 (23)	42 (44)	0.002

Chi-square test and the Mann−Whitney U test were used for comparison

Chronic kidney disease was defined as eGFR < 60 ml/min/1.73 m2. Missing values: a, *n* = 5; b, *n* = 4, c = 12, d = 6

*MRI* magnetic resonance imaging, *TEE* transesophageal echocardiography, *ESUS* embolic stroke of undetermined source, *NIHSS* National Institute Health stroke scale, *LDL-C* low-density lipoprotein cholesterol, *HDL-C* high-density lipoprotein cholesterol, *eGFR* estimated Glomerular Filtration Rate, *BNP* brain natriuretic peptide, *PVH* Periventricular hyperintensity, *DSWMH* deep and subcortical white matter hyperintensity

**Table 2:** Multiple logistic regression analysis predicting a performance of TEE in ESUS

Variables	OR	95% CI	*P*
Age	0.99	0.96–1.02	0.544
Male gender	1.98	0.98–4.01	0.057
Chronic kidney disease	0.72	0.36–1.45	0.358
NIHSS on admission	0.93	0.88–0.98	0.008
Hemoglobin A1c	1.09	0.80–1.49	0.579
BNP	1.00	0.999–1.00	0.215
D-dimer	0.84	0.70–1.00	0.048
Cerebral microbleeds	0.72	0.32–1.62	0.425
PVH, grade ≥ 2	1.2	0.4–3.64	0.748
DSWMH, grade ≥ 2	0.4	0.15–1.30	0.137

*OR* odds ratio, *CI* confidence interval, *TEE* transesophageal echocardiography, *ESUS* embolic stroke of undetermined source, *NIHSS* National Institute Health stroke scale, *BNP* brain natriuretic peptide, *PVH* Periventricular hyperintensity, *DSWMH* deep and subcortical white matter hyperintensity

### Underlying clinical characteristics according to the presence and number of PES in ESUS patients who underwent TEE

In patients who underwent TEE, 20 (17%), 61 (53%), and 34 (30%) patients were classified into none of, one, and ≥ two PES groups, respectively. Table 3 shows age was higher in the ≥ two PES group (69.6 ± 10.8 years, *P* = 0.024), while frequency of diabetes mellitus was lower in the one PES group (34%, *P* = 0.045). LDL-C levels were higher in the none of PES group (128.6 ± 38.9, *P* = 0.039), hemoglobin A1c levels were higher in the ≥ two PES group (6.7 ± 1.4 μg/ml, *P* = 0.022), and D-dimer levels were lower in the none of PES group (0.6 ± 0.3, *P* = 0.002). Age, male gender, diabetes mellitus, and LDL-C, hemoglobin A1c, and D-dimer levels were entered into multinomial multiple logistic regression analysis to determine independent factors according to the number of PES. Compared to the none of PES group, LDL-C (OR: 0.98; 95% CI: 0.96–1.00; *P* = 0.012) and D-dimer (OR: 9.01; 95% CI: 1.00–81.51; *P* = 0.050) were independently associated with the one PES groups, and D-dimer (OR: 9.76; 95% CI: 1.07–88.97; *P* = 0.043) were independently associated with the ≥ two PES groups (Table 4).

**Table 3:** Baseline characteristics and Laboratory and MRI findings according to the presence of potential embolic sources in ESUS patients undergoing TEE

	Potential embolic sources			
Variables	None	One	≥two	***P***
	*n* = 20, 17%	*n* = 61, 53%	*n* = 34, 30%	
**Demographics**				
Age, mean ± SD	62.6 ± 8.7	66.2 ± 13.2	69.6 ± 10.8	0.024
Male gender, n (%)	19 (95)	45 (74)	25 (74)	0.064
Hypertension, n (%)	17 (85)	40 (66)	26 (76)	0.195
Diabetes mellitus, n (%)	12 (60)	21 (34)	19 (56)	0.045
Dyslipidemia, n (%)	18 (90)	44 (72)	27 (79)	0.239
Smoking, n (%)	7 (35)	14 (23)	9 (26)	0.566
Chronic kidney disease, n (%)	4 (20)	16 (26)	10 (29)	0.748
Coronary artery disease, n (%)	1 (5)	2 (3)	1 (3)	0.923
Malignant tumor, n (%)	0 (0)	5 (8)	1 (3)	0.173
History of cerebral infarction, n (%)	4 (20)	7 (11)	9 (26)	0.171
**NIHSS on admission**	2.7 ± 3.2	4.4 ± 6.3	3.6 ± 3.9	0.53
**Laboratory findings**				
LDL-C, mg/dl, a	128.6 ± 38.9	106.8 ± 35.2	119.6 ± 34.2	0.039
HDL-C, mg/dl, a	49.9 ± 17.1	52.3 ± 14.3	51.3 ± 15.1	0.798
eGFR, ml/min	71.9 ± 28.2	71.9 ± 25.1	72.5 ± 24.9	0.962
Hemoglobin A1c, %	6.4 ± 1.2	6.0 ± 0.7	6.7 ± 1.4	0.022
BNP, pg/ml, b	59.3 ± 77.0	86.4 ± 139.3	181.7 ± 593.4	0.739
D-dimer, μg/ml, a	0.6 ± 0.3	1.3 ± 1.5	1.3 ± 1.3	0.002
**MRI findings, n (%)**				
Multiple lesions	10 (50)	38 (62)	18 (53)	0.516
Lesion size > 30 mm in diameter	5 (25)	20 (33)	13 (38)	0.606
Cortical infarction	14 (70)	50 (82)	29 (85)	0.367
Cerebral microbleeds	3 (15)	8 (13)	7 (21)	0.628
PVH, grade ≥ 2	5 (25)	17 (28)	10 (29)	0.941
DSWMH, grade ≥ 2	6 (18)	12 (20)	9 (26)	0.567

Chi-square test and the Mann−Whitney U test were used for comparison

Chronic kidney disease was defined as eGFR < 60 ml/min/1.73 m2. Missing values: a, *n* = 2; b, *n* = 8

*MRI* magnetic resonance imaging, *ESUS* embolic stroke of undetermined source, *TEE* transesophageal echocardiography, *NIHSS* National Institute Health stroke scale, *LDL-C* low-density lipoprotein cholesterol, *HDL-C* high-density lipoprotein cholesterol, *eGFR* estimated Glomerular Filtration Rate, *BNP* brain natriuretic peptide, *PVH* Periventricular hyperintensity, *DSWMH* deep and subcortical white matter hyperintensity

**Table 4:** Multinomial logistic regression analysis predicting factors associated with one and more than two potential embolic sources

	Potential embolic sources					
	One vs. None			≥two vs. None		
Variables	OR	95% CI	*P*	OR	95% CI	*P*
Age	1.01	0.96–1.07	0.689	1.06	0.99–1.13	0.122
Male gender	0.16	0.02–1.54	0.112	0.17	0.02–1.71	0.132
Diabetes mellitus	0.26	0.05–1.24	0.091	0.24	0.04–1.31	0.099
LDL-C	0.98	0.96–1.00	0.012	0.99	0.97–1.01	0.257
Hemoglobin A1c	1.00	0.45–2.22	0.999	2.05	0.98–4.31	0.058
D-dimer	9.01	1.00–81.51	0.05	9.76	1.07–88.97	0.043

*OR* odds ratio, *CI* confidence interval, *LDL-C* low-density lipoprotein cholesterol

### Association of an elevation of D-dimer levels with clinical characteristics and potential embolic sources in ESUS

**Fig. 2:**
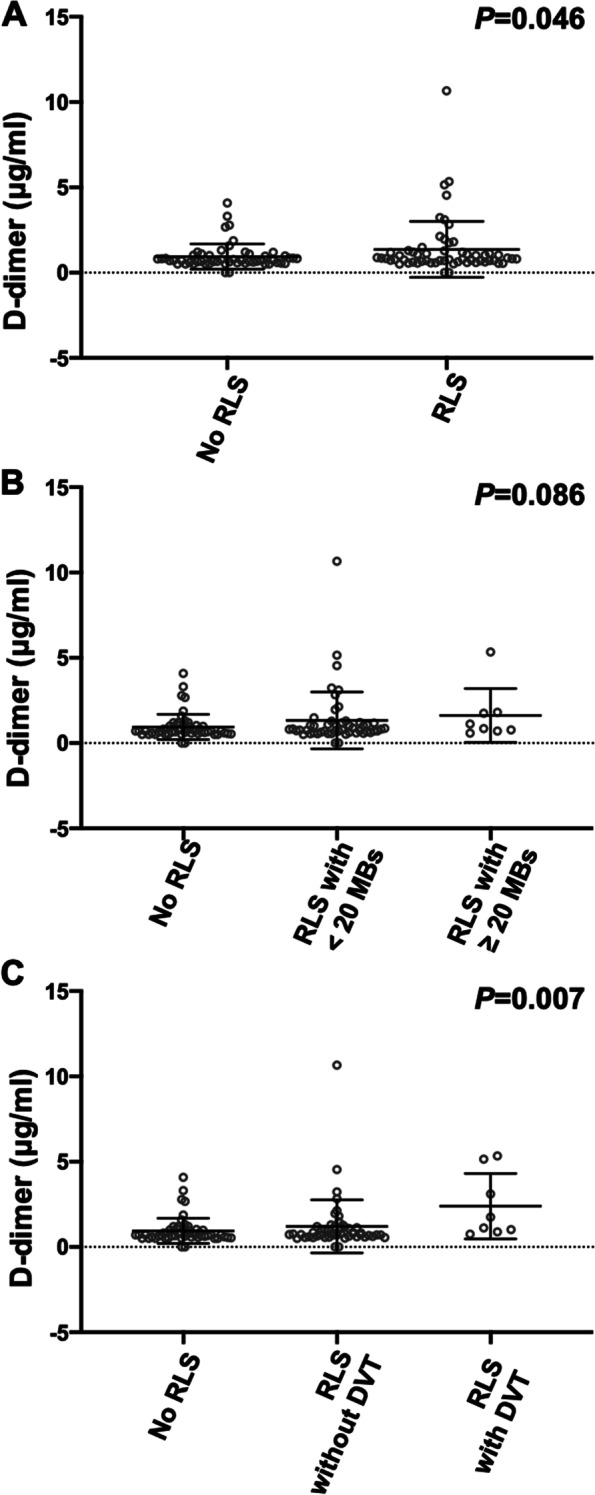
Comparison in D-dimer levels between patients with and without RLS. Distributions of D-dimer levels in patients without RLS (0.94 ± 0.74 μg/ml), and with RLS (1.37 ± 1.64 μg/ml, **A**), and RLS with < 20 MBs (1.33 ± 1.66 μg/ml) and with ≥20 MBs (1.61 ± 1.58 μg/ml, **B**), and RLS without DVT (1.20 ± 1.55 μg/ml) and with DVT (2.99 ± 1.92 μg/ml, **C**). RLS = right-to-left shunt; MBs = microbubbles; DVT = deep venous thrombosis

Next, we analyzed the factors including PES related to an elevation of D-dimer levels. Among PES on TEE, D-dimer concentrations were higher in patients with RLS compared to patients without RLS (1.37 ± 1.64 vs. 0.94 ± 0.74 μg/ml, *P* = 0.046) (Fig. 2). In particular, D-dimer levels were higher in order of RLS with DVT, RLS without DVT, and No-RLS (2.99 ± 1.92 vs. 1.20 ± 1.55 vs. 0.94 ± 0.74 μg/ml, *P* = 0.007), while large shunts more than 20 microbubbles did not show significant difference (Fig. 2). There were no significant differences in the presence of other PES such as ASA, complex aortic plaques, and LAA dysfunction or SEC. 1.0 μg/ml was used as cut-off value to determine an elevation of D-dimer levels [25–27], and ESUS patients who underwent TEE were classified as 75 (66%) with D-dimer levels of ≤1.0 μg/ml and 38 (34%) patients with D-dimer levels of > 1.0 μg/ml. On univariate analysis, patients with D-dimer levels of > 1.0 μg/ml had lower frequency of male gender (63%, *P* = 0.013), and higher frequency of CKD (39%, *P* = 0.017), malignancy (13%, *P* = 0.010), and history of cerebral infarction (29%, *P* = 0.026) (Table 5). In 6 patients with malignancy, 3 patients had active cancer (3 patients had RLS, and D-dimer levels of > 1 μg/ml). Presence of RLS (66%, *P* = 0.040), and PES with DVT (16%, *P* = 0.013) were significantly related to D-dimer > 1.0 μg/ml, which were separately entered to different multiple logistic regression models (Model A and B) because these were covariates. On multiple logistic regression analysis, RLS (OR: 4.62; 95% CI: 1.46–14.61; *P* = 0.009; Model A), and RLS without DVT (OR: 3.90; 95% CI: 1.20–12.70; *P* = 0.024; Model B) and RLS with DVT (OR: 13.94; 95% CI: 1.77–109.99; *P* = 0.012; Model B) were independently associated with D-dimer levels of > 1.0 μg/ml (Table 6).

**Table 5:** Baseline characteristics and Laboratory and MRI findings according to the plasma D-dimer levels in ESUS patients undergoing TEE

	Total	D-dimer levels		
Variables		≤ 1.0 μg/ml	> 1.0 μg/ml	***P***
	*n* = 113	*n* = 75, 66%	*n* = 38, 34%	
**Demographics**				
Age, y	66.9 ± 11.8	66.3 ± 10.5	68.0 ± 14.3	0.153
Male gender, n (%)	87 (77)	63 (84)	24 (63)	0.013
Hypertension, n (%)	82 (73)	57 (76)	25 (66)	0.25
Diabetes mellitus, n (%)	52 (46)	35 (47)	17 (45)	0.846
Dyslipidemia, n (%)	88 (78)	58 (77)	30 (79)	0.846
Smoking, n (%)	29 (26)	21 (28)	8 (21)	0.424
Chronic kidney disease, n (%)	29 (26)	14 (19)	15 (39)	0.017
Coronary artery disease, n (%)	4 (4)	2 (3)	2 (5)	0.492
Malignant tumor, n (%)	6 (5)	1 (1)	5 (13)	0.01
History of cerebral infarction, n (%)	20 (18)	9 (12)	11 (29)	0.026
**NIHSS on admission**	3.9 ± 5.3	3.2 ± 4.2	5.1 ± 6.8	0.084
**Laboratory findings**				
LDL-C, mg/dl, a	114.7 ± 36.6	114.7 ± 36.6	114.8 ± 37.2	0.997
HDL-C, mg/dl, a	51.6 ± 14.9	53.2 ± 15.9	48.5 ± 12.2	0.123
eGFR, ml/min	72.1 ± 25.3	75.8 ± 21.9	64.7 ± 29.8	0.052
Hemoglobin A1c, %	6.3 ± 1.1	6.4 ± 1.2	6.1 ± 0.8	0.353
BNP, pg/ml, b	110.9 ± 342.7	68.8 ± 118.9	196.1 ± 567.7	0.058
**MRI findings, n (%)**				
Multiple lesions	66 (58)	44 (59)	22 (58)	0.937
Lesion size > 30 mm in diameter	38 (34)	21 (28)	17 (45)	0.075
Cortical infarction	92 (81)	61 (81)	31 (82)	0.975
Cerebral microbleeds	18 (16)	12 (16)	6 (16)	0.977
PVH, grade ≥ 2	32 (28)	20 (27)	12 (32)	0.584
DSWMH, grade ≥ 2	27 (24)	17 (23)	10 (26)	0.667
**Potential embolic sources on TEE, n (%)**				
RLS	59 (52)	34 (45)	25 (66)	0.04
Degree of shunt				0.116
Small to Intermediate	51 (45)	30 (40)	21 (55)	
Large	8 (7)	4 (5)	4 (11)	
Comorbidity with DVT				0.013
RLS without DVT	51 (45)	32 (43)	19 (50)	
Both RLS and DVT	8 (7)	2 (3)	6 (16)	
Atrial septal aneurysm	8 (7)	4 (5)	4 (11)	0.322
Aortic arch plaques	53 (47)	35 (47)	18 (47)	0.944
LAA dysfunction or SEC	9 (8)	6 (8)	3 (8)	0.984

Chi-square test and the Mann−Whitney U test were used for comparison

Chronic kidney disease was defined as eGFR < 60 ml/min/1.73 m2. Missing values: a, *n* = 2; b, *n* = 7

*MRI* magnetic resonance imaging, *ESUS* embolic stroke of undetermined source, *TEE* transesophageal echocardiography, *NIHSS* National Institute Health stroke scale, *LDL-C* low-density lipoprotein cholesterol, *HDL-C* high-density lipoprotein cholesterol, *eGFR* estimated Glomerular Filtration Rate, *BNP* brain natriuretic peptide, *PVH* Periventricular hyperintensity, *DSWMH* deep and subcortical white matter hyperintensity, *RLS* right-to-left shunt, *DVT* deep venous thrombosis, *LAA* left atrial appendage, *SEC* spontaneous echo contrast

**Table 6:** Multiple logistic regression analysis predicting an elevation of plasma D-dimer levels

Variables	OR	95% CI	*P*
*Model A*			
Male gender	0.48	0.15–1.52	0.21
Chronic kidney disease	1.63	0.34–7.91	0.541
Malignant tumor	6.25	0.59–66.76	0.129
History of cerebral infarction	3.63	1.03–12.84	0.046
NIHSS on admission	1.08	0.98–1.18	0.111
eGFR	0.97	0.94–1.00	0.087
BNP	1	1.00–1.01	0.403
Lesion size > 30 mm in diameter	2.13	0.75–6.05	0.155
RLS	4.62	1.46–14.61	0.009
*Model B*			
Male gender	0.49	0.15–1.61	0.242
Chronic kidney disease	1.66	0.34–8.19	0.536
Malignant tumor	6.21	0.56–69.27	0.138
History of cerebral infarction	3.55	1–12.57	0.05
NIHSS on admission	1.07	0.98–1.18	0.153
eGFR	0.97	0.94–1.01	0.093
BNP	1.00	1.00–1.01	0.348
Lesion size > 30 mm in diameter	2.04	0.71–5.91	0.188
RLS			
Without DVT	3.9	1.20–12.70	0.024
With DVT	13.94	1.77–109.99	0.012

*OR* odds ratio, *CI* confidence interval, *eGFR* estimated Glomerular Filtration Rate, *BNP* brain natriuretic peptide, *RLS* Right-to-left shunt, *DVT* deep venous thrombosis

## Discussion

The present study explored the factors related to performance of TEE, and the association of PES demonstrated on TEE with D-dimer levels in ESUS patients. Our results showed that ESUS patients undergoing TEE displayed low NIHSS scores and low D-dimer levels compared to ESUS patients not undergoing TEE. In patients undergoing TEE, D-dimer levels were higher in the one PES group and ≥ two PES group than those in the none of PES group. Furthermore, RLS, with and without DVT, were independently associated with D-dimer levels of > 1.0 μg/ml in ESUS.

TEE is useful to comprehensively assess sorts of PES and clarify stroke pathogenesis, and a gold standard to diagnose RLS. However, TEE is not mandatory for the diagnosis of ESUS [4], and limited studies have been conducted in ESUS [9, 10]. TEE is a semi-invasive method, and not a few patients can not be performed with severe neurological symptoms, comorbidity such as pneumonia and congestive heart failure, and patients’ refusal. Not TEE-conducted cases in our cases displayed higher NIHSS score, and furthermore these cases displayed higher D-dimer levels. It is suggested that severe stroke disability might result in thrombotic formation [28]. Current results identified that the clinical characteristics of TEE performance for ESUS by stroke physicians from a single-center experience.

The current data demonstrated that D-dimer levels were higher in the one and greater than two PES groups than those in none of PES group. Emerging data indicated that elevation of D-dimer is related to newly onset of DVT, malignancy, and hypercoagulable states [16, 29]. As for DVT, Lippi et al. demonstrated that patients with DVT displayed 2.5 μg/ml in D-dimer levels among 1647 patients who admitted in emergency department [30]. Among stroke subtype, a systematic review showed that the cut-off value of D-dimer levels for cardioembolic stroke was high to range from 0.3 to 2.0 μg/ml [31]. Atrial cardiopathy and cardiac calcification, not evaluated in the current study, were related to new onset of AF in ESUS [32, 33]. Importantly, atrial cardiopathy might promote blood stasis and thrombogenesis, and thus raise D-dimer levels [34]. In aortogenic embolic stroke, the extent of D-dimer elevation limited in mild levels (≈1.0 μg/ml) [35]. Meanwhile, ischemic stroke with active cancer showed further high D-dimer levels of ≥5 μg/ml, and the association of occult cancer with D-dimer in ESUS has been focused [31]. In our study, RLS, with or without DVT, was shown to link with elevations of D-dimer levels in ESUS. Since D-dimer reflects the result of not only thrombus formation but also secondary fibrinolysis, and it is possible that the formed thrombus has dissolved after onset of paradoxical brain embolism (PBE) which might have failed to recognize DVT. A previous study by Nezu et al. demonstrated that high D-dimer levels were related to long-term outcomes (recurrent stroke or all-cause mortality) in patients with cryptogenic stroke, especially in those with RLS [36]. They speculated that presence of known or occult cancer might be cause of recurrent stroke or all-cause mortality [36]. This is the first study to indicate the clinical usefulness of measuring D-dimer levels to explore the potential embolic etiologies in ESUS. However, RLS could be incidental rather than embolic origins especially in patients with ≥two PES. Furthermore, left atrial diameter and volume index, cardiac calcification, and occult cancer were not evaluated, and thus one could not exclude the possibility that other potential stroke etiologies including atrial cardiopathy, paroxysmal AF, and cancer-related hypercoagulability existed and were related to elevation of D-dimer levels in the present study.

This study has some limitations. First, the data from the current study were derived from a single center, and the number of patients is quite small, especially in ESUS patients with TEE and D-dimer levels > 1.0 μg/ml. Second, some patients were taking medications included statins, antithrombotic drugs, and anti-hypertensive agents prior to stroke onset, which might affect the baseline characteristics and laboratory data such as LDL-C, BNP, and D-dimer. Moreover, atrial cardiopathy, cardiac calcification, and occult cancer, which might be related to D-dimer levels, were not investigated during hospitalization. This study was retrospective in nature so that these effects were not assessed in the current study. Third, although three experienced stroke physicians decided to perform TEE in the conference considering the safety and avoiding complications according to the American Society of Echocardiography Guidelines and Standards [18], TEE was done without randomization, and thus there might have been the selection bias. Moreover, clinical characteristics between ESUS patients with and without TEE were different, which raised an issue regarding the generalizability of the results to the entire ESUS population.

## Conclusion

Our results showed that more than half of ESUS patients received TEE examinations, with characteristics with mild neurological symptoms and lower D-dimer levels. In ESUS with TEE investigations, RLS was related to elevation of D-dimer. Although the current study indicated that D-dimer could possibly be a surrogate marker for PBE among ESUS, RLS was incidental rather than embolic origins in some patients, and other potential stroke etiologies not evaluated in the current study might be associated with elevation of D-dimer levels. Further study with large sample size is warranted.

## Data Availability

The datasets used and analyzed during the current study are available from the corresponding author upon reasonable request.
